# The Common Task

**Published:** 1907-10-12

**Authors:** 


### THE COMMON TASK.

### The Roof Hospital.

A very interesting suggestion has been made by Dr. James Dunbar-Brunton that roof gardens shall be made a regular feature of city life in crowded localities, and that these shall be supplemented by roof hospitals where little sufferers from hip disease, rickets, and kindred complaints should gain the air and sunlight essential to their recovery. The project is explained in a little pamphlet, "Better Hygiene for the Children of the Poor" (Scientific Press, price 6d.), and will, we hope, bear fruit in endeavours to carry out some part of the scheme experimentally in the East End. The theory is that flat roofs, protected by a parapet-retaining wall, should be secured on all new buildings, where children could play in safety, each roof having a covered part as a shelter from rain. The roof hospitals "could be built of frames securely fastened to the roof, and have as much glass in their structure as possible. The roof would have large glass fanlights protected by blinds, which could be pulled down. . . ." They could contain two wards besides accommodation for the nurse, lavatories, etc., and should be under the inspection of the medical officer of health. While cordially agreeing with the desirability of the roof garden, we demur to the multiplication of small urban hospitals. A child ill enough to be under constant nursing ought to be got into the country, and no height above the road will supply the place of a pure quality in the air.

### The Proportion of Sisters in Hospitals.

It is curious that there is no agreement in hospitals respecting the number of sisters it is desirable to appoint in proportion to other members of the staff. The proportion varies in the large London hospitals from one in 14 at the Middlesex, and one in 11 at the Royal Free Hospital, to one in six at the London, at St. George's, and at the Charing Cross Hospitals. In the provinces the proportion is from one in seven at the Leeds General Infirmary to one in five at the Bristol General Infirmary. At the Edinburgh Royal Infirmary the proportion is one in seven. At the Dublin Meath Hospital it is one in nine. Nothing makes greater pressure in hospital than a shortage of sisters. Indeed, where the matron is obviously overburdened it is a very doubtful advantage to supplement with probationers. It merely means more people to be taught. One sister is of more value than several probationers, and though from the point of view of salaries it may seem that expense is incurred by increasing the number of sisters, it is in reality a less costly step than to add unskilled workers who, though they receive no pay, will cost the hospital from first to last quite £50 a year during their training. The proportion of occupied beds which falls to the sisters varies also considerably from an average of ten at the London Hospital to an average of 20 at King's College Hospital. Variations in construction and organisation are responsible for these distinctions, which go far to show that no stereotyped system of administration has yet been acknowledged as the best.

### New Issue of the Midwives Roll.

The recent issue of the Midwives Roll contains a total of 24,338 names, of which number 2,406 appear in virtue of having passed the examination of the Central Midwives Board. It is matter for the deepest regret that the number of Poor-law infirmaries recognised as training schools by the Central Midwives Board is but six in London, eight for the rest of England, one in Wales, two in Scotland (both in Glasgow), and one in Ireland. Whatever tends to differentiate the Poor-law training schools from voluntary establishments is a drawback to their efficiency, and tends to have an adverse influence on the class of probationer attracted to the work. Poor-law infirmaries must always have in training more nurses than are required for the vacancies in the service, and their success must largely depend on their ability to fit their pupils for good appointments. To this end recognised midwifery training is a factor the importance of which it is impossible to exaggerate. It is against the best interests alike of the poor and of the nurses that an attempt should be made to dissociate the Poor-law nurse from obligations imposed on persons trained in voluntary institutions, and we believe that it is only a question of time before this is clearly understood by the Local Government Board, and their present attitude towards the Central Midwives Board is abandoned.

